# Toward standardized brain tumor tissue processing protocols in neuro-oncology: a perspective for gliomas and beyond

**DOI:** 10.3389/fonc.2024.1471257

**Published:** 2024-09-23

**Authors:** Analiz Rodriguez, Manmeet S. Ahluwalia, Chetan Bettegowda, Henry Brem, Bob S. Carter, Susan Chang, Sunit Das, Charles Eberhart, Tomas Garzon-Muvdi, Costas G. Hadjipanayis, Cynthia Hawkins, Thomas S. Jacques, Alexander A. Khalessi, Michael W. McDermott, Tom Mikkelsen, Brent A. Orr, Joanna J. Phillips, Mark Rosenblum, William J. Shelton, David A. Solomon, Andreas von Deimling, Graeme F. Woodworth, James T. Rutka

**Affiliations:** ^1^ Department of Neurosurgery, College of Medicine, University of Arkansas for Medical Sciences, Little Rock, AR, United States; ^2^ Department of Medical Oncology, Miami Cancer Institute, Baptist Health South Florida, Miami, FL, United States; ^3^ Department of Neurosurgery, Johns Hopkins Hospital, Baltimore, MD, United States; ^4^ Department of Neurosurgery, Massachusetts General Hospital and Harvard Medical School, Boston, MA, United States; ^5^ Division of Neuro-Oncology, Department of Neurosurgery, University of California San Francisco, San Francisco, CA, United States; ^6^ Division of Neurosurgery, St. Michael’s Hospital, University of Toronto, Toronto, ON, Canada; ^7^ Department of Pathology, Johns Hopkins University School of Medicine, Baltimore, MD, United States; ^8^ Department of Neurosurgery, Emory University, Atlanta, GA, United States; ^9^ Department of Neurological Surgery, University of Pittsburgh School of Medicine, Pittsburgh, PA, United States; ^10^ Division of Pathology, Hospital for Sick Children, Toronto, ON, Canada; ^11^ Developmental Biology and Cancer Programme, UCL GOS Institute of Child Health and Department of Histopathology, Great Ormond Street Hospital NHS Foundation Trust, London, United Kingdom; ^12^ Department of Radiology and Neurosciences, Don and Karen Cohn Chancellor’s Endowed Chair of Neurological Surgery, University of California, San Diego, San Diego, CA, United States; ^13^ Division of Neurosurgery, Miami Neuroscience Institute, Miami, FL, United States; ^14^ Department of Neurosurgery, Hermelin Brain Tumor Center, Henry Ford Health System, Detroit, MI, United States; ^15^ Department of Pathology, St. Jude Children’s Research Hospital, Memphis, TN, United States; ^16^ Department of Neurological Surgery, University of California, San Francisco, San Francisco, CA, United States; ^17^ Neuropathology Division, Department of Pathology, University of California, San Francisco, San Francisco, CA, United States; ^18^ Department of Neurosurgery, Omics Laboratory, Hermelin Brain Tumor Center, Henry Ford Health System, Detroit, MI, United States; ^19^ Division of Neuropathology, Department of Pathology and Helen Diller Family Comprehensive Cancer Center, University of California, San Francisco, San Francisco, CA, United States; ^20^ Department of Neuropathology, Institute of Pathology, Ruprecht-Karls-University of Heidelberg, Heidelberg, Germany; ^21^ Department of Neurosurgery, University of Maryland School of Medicine, Baltimore, MD, United States; ^22^ Division of Neurosurgery, Chair Emeritus, Hospital for Sick Children, Toronto, ON, Canada

**Keywords:** brain tumors, biobank, tissue processing, precision medicine, gliomas

## Abstract

Implementation of standardized protocols in neurooncology during the surgical resection of brain tumors is needed to advance the clinical treatment paradigms that use tissue for diagnosis, prognosis, bio-banking, and treatment. Currently recommendations on intraoperative tissue procurement only exist for diffuse gliomas but management of other brain tumor subtypes can also benefit from these protocols. Fresh tissue from surgical resection can now be used for intraoperative diagnostics and functional precision medicine assays. A multidisciplinary neuro-oncology perspective is critical to develop the best avenues for practical standardization. This perspective from the multidisciplinary Oncology Tissue Advisory Board (OTAB) discusses current advances, future directions, and the imperative of adopting standardized protocols for diverse brain tumor entities. There is a growing need for consistent operating room practices to enhance patient care, streamline research efforts, and optimize outcomes.

## 1 Introduction

The average annual age-adjusted incidence rate of primary brain tumors (malignant and non-malignant) is 24.71 per 100,000 population. The most common malignant and non-malignant primary brain tumor are glioblastoma and meningioma respectively (1). Secondary metastatic brain tumors can develop in approximately 20% of all patients with cancer making metastatic brain tumors more common than primary brain tumors (2). Surgical resection or biopsy of brain tumors is routinely performed for diagnosis as well as treatment in many circumstances. The fifth edition of the World Health Organization Classification of primary brain tumors released in 2021 has significant updates which require molecular characterization for tumor classification (3). These classifications require further assays beyond standard histopathology and thereby warrant more tissue availability. Besides providing diagnostic information, the tissue obtained from these surgical procedures can also be used for translational research purposes such as the development of preclinical models and provide pharmacodynamic information in early phase clinical trials such as Phase 0 window of opportunity trials (4, 5). To date there is no standard operating procedure for neurosurgeons during surgical resection of brain tumors. With the advent of fresh tissue being used for various clinical assays and models, neurosurgeons need to provide feasible guidelines that can be implemented easily at various centers. While recent recommendations from the Response Assessment in Neuro-Oncology (RANO) consortium provide a pivotal framework for diffuse gliomas, the Oncology Tissue Advisory Board (OTAB) aims to extend these protocols to encompass all brain tumors for comprehensive and unified patient care, with more specificity to the procedures of tissue procurement.

## 2 Glioma surgery recommended guidelines

Treatment of newly diagnosed diffuse glioma (DG) involves surgery, radiation and systemic pharmacotherapy (6). Surgical resection remains the first line of treatment and is necessary for diagnosis and molecular characterization of the tumor to guide future therapy. It plays a relevant role during the initial diagnosis and recurrent disease, aiding to tailor treatment regimens and assist with the development of novel drugs (7). In *The Lancet Oncology*, Karschnia and colleagues report recommendations from the Response Assessment in Neuro-Oncology (RANO) consortium on the need for standardized protocols following surgical resection for accurate clinical tumor evaluation as well as prospective biobanking for research purposes (8). The framework proposed requires neurosurgeons to consider surgical resection trajectories to provide tissue from multiple spatial regions given the heterogeneity of DG. “Geo-tagging” tumor samples to record the location of samples within the tumor requires integration with neuronavigation platforms during the surgical resection (9). Furthermore, the recommendations include sample processing immediately in the operating room (OR) following resection which requires significant infrastructure (4). These advancements in standardization are necessary given the need for more accurate characterization of the tumor’s biology when representative tissue is obtained aiding in diagnosis, prognosis, and treatment.

The current landscape of neurosurgical ORs exhibits a spectrum of practices, lacking a cohesive standard across institutions which can lead to variability in tissue sample retrieval and integrity. This is reflected by the lack of standardization towards tissue handling, with no consensus on ideal conditions for surgical tissue preservation directed towards molecular/pathologic diagnosis and translational research purposes (10). Inadequate infrastructure and OR personnel are an additional contributing factor for difficulties in tissue preservation, collection, and transportation (10). Ideally, surgical specimens should be processed within 30 minutes of tissue recollection, especially for ‘omics’ analyses. This is crucial due to the changes in gene and protein expression caused by external factors following tissue removal like ischemia (11–13). Furthermore, the selection of surgical tools for tissue resection is pivotal for specimen preservation. For example, certain tools, such as the ultrasonic aspirator, can thermally heat tissue and lead to direct tissue damage by causing apoptosis of cell membranes. Nonetheless, some studies have reported some degree of success using ultrasonic aspirators. For example, Day et al., used a cavitron ultrasonic surgical aspirator (CUSA) to obtain 48 samples. The viability of the recollected tissues was high enough to conduct *ex vivo* cell cultures, cytometric analysis, and patient-derived xenograft intracranial animal models (14). Similar studies have successfully established brain tumor cell lines obtained from CUSA samples, with viabilities reported for isolated cells ranging from 67-82% (15, 16). Other ultrasonic aspirators, like the Söring ultrasonic aspirator and Stryker Sonopet, are also available and can be used for research purposes. In cases where surgical resources are limited, tumor collection chambers can be made with easily accessible and economical devices (17). For instance, in a study done by Ruparelia et al., a sterile and disposable mucous extractor device was employed for collecting brain tumors samples, facilitating tissue recollection (18).

**Figure 1:**
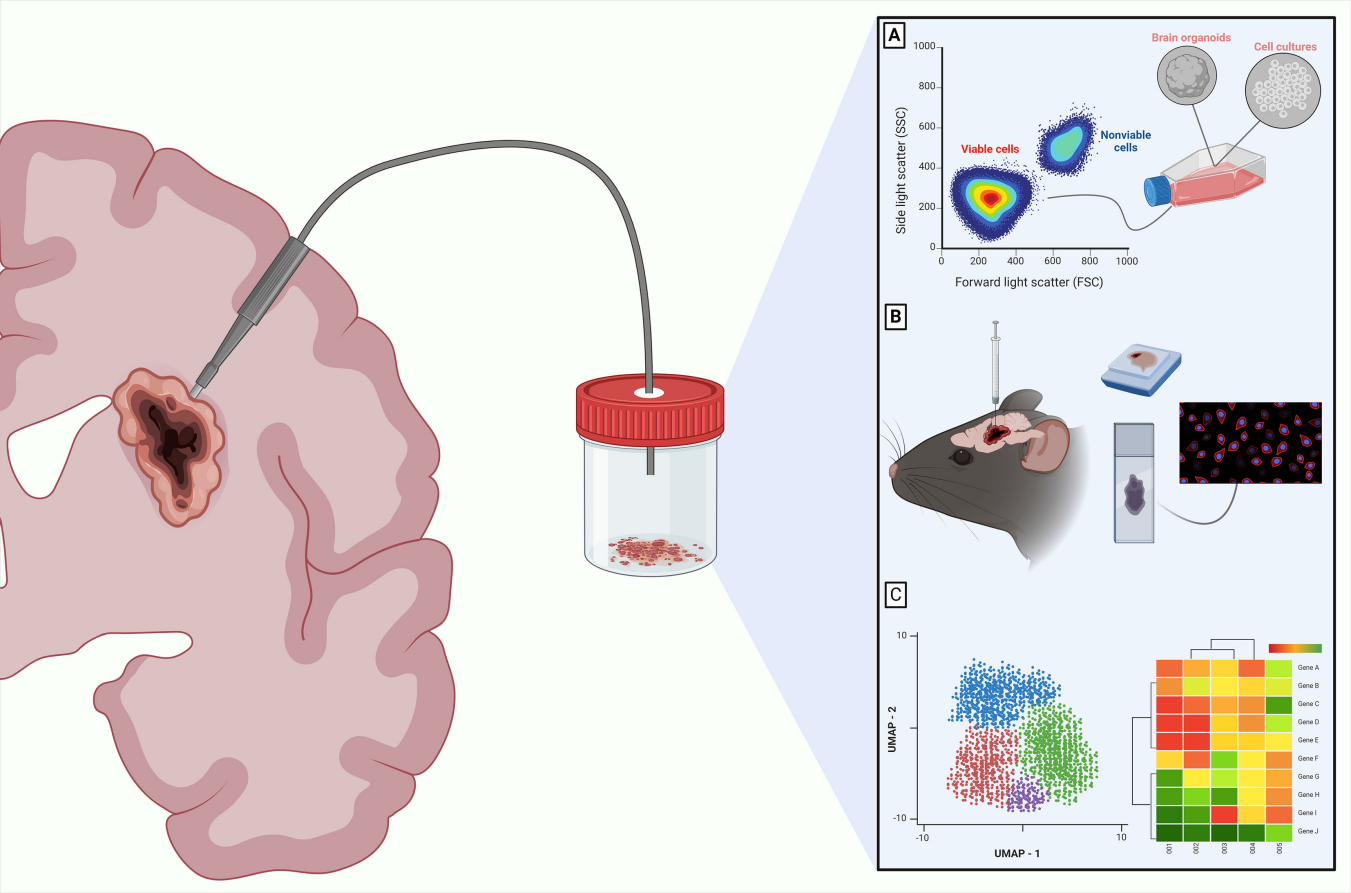
Downstream analysis for CNS tumors in viable resected tissues. The importance of employing intraoperative surgical tools that maintain high cellular viability is highlighted by different translational applications for CNS tumors. These applications include culturing cells **(A)**, developing preclinical models using patient derived xenografts **(B)**, and performing downstream sequencing with multi-omics analyses such as the Uniform Manifold Approximation and Projection (UMAP) or genomic heatmaps from transcriptomics data **(C)**. These are just some of the many options available. Created with Biorender.com.

On the other hand, surgeons can consider using non-destructive surgical tools that can automatically, biologically, preserve tissue in a sterile environment, while isolating it from atmospheric conditions. Different instruments are available for this purpose. For example, the NICO Myriad/APS System (NICO Corporation, Indianapolis, IN) is a multi-functional, non-ablative, tissue resection device that uses a guillotine-like cutting aperture and variable suction to grasp and cut small, targeted blocks of architecturally intact tissue. With this system, several cubic millimeters can be harvested each minute. These tissue blocks are then biologically maintained in chilled aqueous non-oxygenated environment as they are captured within a sterile container, which can then be used for future downstream assays (**Figure 1**) (10, 19). Various handheld devices are used intraoperatively to assist in surgical resection and comparison of brain tumor tissue collection parameters, such as tumor cell *ex vivo* viability, is warranted and ongoing (20). There is minimal information for neurosurgeons on how their surgical methods can affect biospecimen quality. In summary, preferred intra-operative systems should be simplified, standardized, site annotated, and able to obtain the maximum amount of tissue possible with minimal perturbation during acquisition. Furthermore, tissue specimens should be available for processing as rapidly as possible while being maintained in physiological conditions.

Another major hurdle for neurosurgeons during surgical resection of DG is the inability to differentiate tumor from normal brain. Fluorescence-guided surgery with 5-aminolevulinic acid (5-ALA; Gleolan ^®^) is often used during the resection of DG to augment the ability to achieve maximal surgical resection (21, 22). 5-ALA requires that the surgeon uses a blue filter (400nm wavelength) to visualize protoporphyrin IX (PpIX) uptake within the tissue. DG cells metabolize 5-ALA to fluorogenic PpIX via the heme synthesis pathway and the fluorescence is highly sensitive, specific, and accurate for labeling of malignant cells and not normal brain or necrosis making the agent an ideal adjunct to neurosurgeons. However, not all institutions have optical platforms with the blue filter available and therefore national neurosurgical standardization for the utilization of 5-ALA has yet to occur. Recently 5-ALA has also been used for the resection of other tumor types such as meningiomas (23).

## 3 Intraoperative considerations for diverse brain tumors

Brain tumors can be intra-axial or extra-axial and have various tissue properties. Surgical approaches for tumors require consideration of the tumor location. Along with traditional craniotomy techniques, endonasal endoscopic surgery can be used for various pathologies such as pituitary neuroendocrine tumors, craniopharyngiomas, chordomas, osteosarcomas, and skull base meningiomas. The consistency of these tumors can be quite varied and intraoperative surgical decision-making can be dependent on the firmness of tumor encountered. Efforts are being made to develop preoperative imaging tools to better guide neurosurgeons in predicting the consistency of the tumor (24). Due to many constraints such as small size, possible firm consistency, and difficult access, tissue obtained from tumors resected by endonasal approaches are typically solely used for diagnostic pathology and not for the development of preclinical research models. There is a dearth of preclinical research models for brain tumor pathologies such as pituitary neuroendocrine tumors and craniopharyngiomas. Transgenic mouse models are available for some of these pathologies but few patient-derived models are available (25). Intraventricular tumors have similar considerations and limitations. Currently the cancer cell encyclopedia has 117 primary brain tumor cell lines but most are DG with only 19 embryonal pediatric brain tumors and 4 meningiomas (26).

Advancements in the understanding of cancer biology have led to the development of novel targeted therapies based on precise and characteristic genotypic aberrations among tumors, including those found in brain metastases (BM). In these tumors, comprehension of their nature is crucial, as they present different therapeutic barriers, such as genotypical divergence from their primary origin (27, 28). Additionally, key biological mechanisms intrinsic to BM carcinogenesis have major therapeutic implications, such as the brain invasion cascade, blood brain barrier dynamics, genomic mediators of CNS tropism, and the brain tumor microenvironment (29). This highlights the importance of tissue acquisition for guiding treatment and encouraging clinical trial enrollment. Therefore, characterizing key components such as the genomic landscape of individual tumors makes it possible to discern clinically and genomically distinct features among them, which may be actionable through personalized treatments (30). This is especially relevant in BM, given that commonly used therapies in CNS tumors, such as cytotoxic chemotherapies, have demonstrated exceedingly low response rates. For example, in melanoma BM, temozolomide has shown clinical response rates of 3-7% in prospective studies (31).

Pediatric brain tumors are the most common type of solid cancer and are the leading cause of cancer-associated death in children. Medulloblastoma is the most common malignant pediatric brain tumor and there are 4 distinct molecular subtypes (32). Pediatric high-grade gliomas, ependymomas, and atypical teratoid rhabdoid tumors are tumors that also can have aggressive clinical courses (33). Given the heterogeneity of these tumors, no standardized protocols exist for the surgical resection of pediatric brain tumors. Certain tumors are in locations wherein the risks of neurological morbidity are very high and therefore surgery is not a consideration. However, tissue acquisition, even posthumously, has been critical for the development of therapies for lethal pediatric brain tumors. Through tissue acquisition of Diffuse intrinsic pontine glioma (DIPG) and other H3K27M-mutated diffuse midline gliomas (DMGs), a targetable disialoganglioside GD2 was identified and ultimately used in a Phase 1 clinical trial for patients with this devastating disease (34). This example sheds light on the critical importance of tissue acquisition for therapeutic development.

## 4 OTAB proposed framework

**Figure 2:**
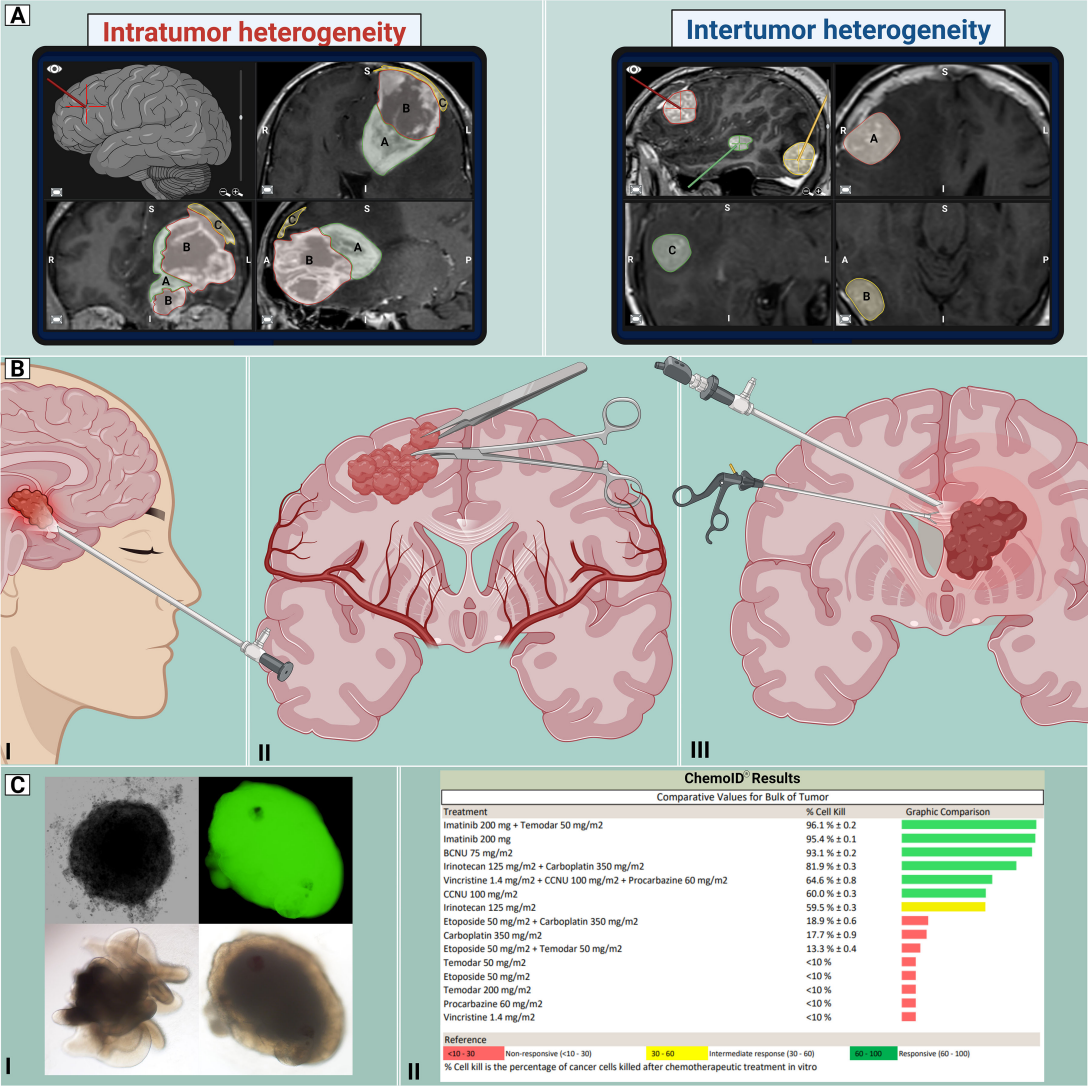
Proposed workflow for CNS tumors. Standardization of the management of CNS tumors enables better unification of neurosurgical practice by establishing the best current approaches for managing such diverse pathologies. By acknowledging the intricate nature and diversity of CNS tumors, including intratumor and intertumor heterogeneity **(A)**, an array of cranial approaches **(B)** can be employed, including endonasal endoscopic (I), open “classic” craniotomy (II) and endoscopic intraventricular (III) techniques for tumor removal. With the use of precision medicine **(C)**, the retrieved tissue could be utilized for developing clinically relevant models, such as organoids and spheroid tumor models (I), and functional assays (II), demonstrating susceptibility patterns to a variety of chemotherapeutic drugs. This information could guide physicians in determining appropriate treatments. Created with Biorender.com.

In the era of precision medicine, brain tumor biobanking can lead to significant advances in further understanding pathophysiology, developing therapy, and providing clinically relevant models (35). Brain tumors exhibit considerable heterogeneity and standardized protocols must navigate the challenges posed by varying tumor characteristics. A critical aspect of standardization involves unifying operating room practices for all neurosurgical interventions. From preoperative imaging to postoperative tissue handling, protocols should transcend tumor types, fostering a seamless and standardized workflow. These workflows need to integrate perspectives from radiologists, neurosurgeons, and pathologists. During preoperative planning, considerations can be made about locations within the tumor that will be harvested for tissue sampling. Furthermore, in cases where multiple tumors are removed at one time, annotation of the location and tumor are needed. Considerations of geolocations allow for studies of inter and intra-tumor heterogeneity (**Figure 2A**).

Brain tumors that are accessed endoscopically (either via transnasal or intraventricular approaches) can pose considerable limitations. Due to the long operative corridor needed for these approaches, some surgical tools are not able to be used in these limited working spaces. The surgeon also needs to consider having equipment available that ‘traps’ tissue obtained from surgical suction canisters to prevent tissue wasting and optimize tissue harvesting from all locations (**Figure 2B**). During surgical resection, it is critical to obtain diagnostic tissue and therefore collaboration between the surgeon and pathologist are warranted intraoperatively. Multidisciplinary crosstalk is needed to assure that the tissue needed by the pathology team is not compromised. Analysis of intraoperative samples for frozen sectioning is often employed to assess the presence of diagnostic tissue. The generation of advanced preclinical models and functional assays oftentimes requires fresh tissue of high quality without necrotic regions (**Figure 2C**). The pathology team can aid the neurosurgeon in choosing the best tissue for these various applications. Preoperative input from the neuro-oncologist is also needed to determine patients that can benefit clinically from these assays. Therefore, intraoperative tissue sampling requires a multidisciplinary approach and has immense translational capabilities.

## 5 Current challenges

Numerous challenges arise when establishing high-quality tissue biobanks, especially in under resourced institutions. One of the main barriers to successful biobanking is ensuring that healthcare workers understand its value and thereby are motivated to support these efforts. There is a dependency on the knowledge and attitude of healthcare providers for ensuring successful biobanking as lack of support has been as a major hindrance to obtaining high-quality samples (36). We therefore advocate for the formation of consortiums to provide necessary education and aid in the formation of sustainable standard operating procedures that can be implemented at various organizations. Insights from international biobanking networks can help identify and address barriers (37, 38). For centers without the necessary infrastructure on site, samples could be potentially sent to a larger center. Furthermore, big data integration with biobanking to further precision medicine strategies can allow for patients from multiple centers to benefit.

Biobanking efforts must take into account the ethical challenges involved in using biospecimens for research. The legal landscape surrounding biospecimen utilization in biomedical research may change drastically in the near future with legal cases pending which will challenge the scope of research allowed on non-identified specimens in the United States (39). These new regulations will need to be implemented in biobanking protocols with efforts to respect the autonomy and beliefs of donor participants (40).

## 6 Future directions

Recently, fresh tumor tissue from neurosurgical resections has been utilized for emerging clinical protocols. Obtaining real-time diagnostic information during surgical resection has become feasible. During the initial phases of resection, neurosurgeons can submit small tumor samples to undergo rapid genetic sequencing and, in combination with a machine learning platform, can lead to a rapid and accurate tumor diagnosis (41). Advances in diagnostic technologies facilitate the management of CNS tumors. The implementation of cutting-edge systems into surgical workflow overcomes traditional tissue processing barriers and improves diagnostic efficiency by providing rapid and accurate diagnoses. Different studies have demonstrated the benefits of intraoperative technologies, including high-resolution optical devices, such as fiber laser-based and label-free contrast Raman histology, or machine-learning methods, such as deep neural networks for analyzing pathological specimens (41–45). For example, Hollon et al., used stimulated Raman histology (RH) and convolutional neural networks (CNN), trained with over 2.5 million labeled patches from RH, for near real-time diagnosis of 278 tumor patients. The results of this study demonstrated the noninferiority of CNN-RH based diagnosis compared to pathologist-based interpretation of conventional histopathology techniques (e.g., H&E), with accuracies of 94.6% and 93.9%, respectively (45). These technologies enable neurosurgeons to adjust their surgical strategy towards a less or more aggressive resection depending on the brain tumor subtype. Maximal surgical resection is often the goal for DG and other brain tumors, but this must be balanced with the risk of causing neurological morbidity. In this way, intraoperative diagnostic information can aid neurosurgeons in determining the extent of resection goals especially when linked to brain mapping and neuromonitoring techniques.

Another emerging utility for fresh tissue includes functional precision medicine (FPM) wherein patient tissues are directly exposed to standard and novel agents to identify tumor vulnerabilities. In newly diagnosed high-grade glioma (HGG) patients, a FPM assay based on *ex vivo* spheroids generated from surgically resected tissue samples was able to predict temozolomide (standard of care chemotherapy for HGG) responders from non-responders (46). In a recent trial in recurrent HGG patients, physician choice was compared to a glioma stem cell (GSC) guided FPM assay derived from biopsy tissue. The FPM group had longer median survival and lower risk of death demonstrating the feasibility of integrating these protocols into clinical regimens (47). These developing applications of fresh surgical brain tumor tissue specimens highlight the emerging imperative to obtain high-quality fresh tissue. The accuracy of diagnostic and therapeutic interventions is intricately linked to the quality of biological samples, underscoring the importance of a standardized framework.

Finally, the integration of cell-free and circulating tumor DNA in liquid biopsies (e.g., blood or cerebrospinal fluid) complements the genomic profiling of each tumor and serves as an important tool for monitoring circulating biomarkers that could potentially predict disease progression and response to established treatments (48, 49). In such cases, sequencing technologies hold great potential, as their improvement in the detection of these biomarkers will facilitate the integration of disease monitoring throughout the course of the disease.

## 7 Conclusion

In conclusion, multidisciplinary neuro-oncology teams must champion the call for standardized operating room protocols to harness the full potential of these recommendations. Integrating multimodal approaches from different medical teams, starting from initial surgical planning to maximize the resection and viability of malignant tissues. This includes the use of multiple trajectories in relevant geolocations to ensure heterogeneous sampling of the tumors, along with pre-, intra- and post-operative inputs from neuro-oncologists and neuropathologists. This approach would help establish appropriate and personalized treatments based on genomic profiling outputs, with further extrapolation to translational and preclinical models to improve cancer management. By doing so, they not only optimize their workflows and enhance patient safety but also pave the way for advancements in advanced molecular diagnostics and functional precision medicine assays, ushering in a new era of precision neuro-oncology. This unified approach marks a significant stride toward advancing brain tumor patient care.

## Data Availability

The original contributions presented in the study are included in the article/supplementary material. Further inquiries can be directed to the corresponding author.
